# A Cytoplasmic Domain Mutation in ClC-Kb Affects Long-Distance Communication Across the Membrane

**DOI:** 10.1371/journal.pone.0002746

**Published:** 2008-07-23

**Authors:** Gilbert Q. Martinez, Merritt Maduke

**Affiliations:** Department of Molecular and Cellular Physiology and Program in Biophysics, Stanford University. Stanford, California, United States of America; Massachusetts Institute of Technology, United States of America

## Abstract

**Background:**

ClC-Kb and ClC-Ka are homologous chloride channels that facilitate chloride homeostasis in the kidney and inner ear. Disruption of ClC-Kb leads to Bartter's Syndrome, a kidney disease. A point mutation in ClC-Kb, R538P, linked to Bartter's Syndrome and located in the C-terminal cytoplasmic domain was hypothesized to alter electrophysiological properties due to its proximity to an important membrane-embedded helix.

**Methodology/Principal Findings:**

Two-electrode voltage clamp experiments were used to examine the electrophysiological properties of the mutation R538P in both ClC-Kb and ClC-Ka. R538P selectively abolishes extracellular calcium activation of ClC-Kb but not ClC-Ka. In attempting to determine the reason for this specificity, we hypothesized that the ClC-Kb C-terminal domain had either a different oligomeric status or dimerization interface than that of ClC-Ka, for which a crystal structure has been published. We purified a recombinant protein corresponding to the ClC-Kb C-terminal domain and used multi-angle light scattering together with a cysteine-crosslinking approach to show that the dimerization interface is conserved between the ClC-Kb and ClC-Ka C-terminal domains, despite the fact that there are several differences in the amino acids that occur at this interface.

**Conclusions:**

The R538P mutation in ClC-Kb, which leads to Bartter's Syndrome, abolishes calcium activation of the channel. This suggests that a significant conformational change – ranging from the cytoplasmic side of the protein to the extracellular side of the protein – is involved in the Ca^2+^-activation process for ClC-Kb, and shows that the cytoplasmic domain is important for the channel's electrophysiological properties. In the highly similar ClC-Ka (90% identical), the R538P mutation does not affect activation by extracellular Ca^2+^. This selective outcome indicates that ClC-Ka and ClC-Kb differ in how conformational changes are translated to the extracellular domain, despite the fact that the cytoplasmic domains share the same quaternary structure.

## Introduction

Members of the CLC family of chloride channels and transporters are ubiquitously expressed and serve a variety of physiological functions [1], [2]. Mutations in human CLCs give rise to a number of diseases including Bartter's syndrome (ClC-Kb [3]–[7]), myotonia congenita (ClC-1 [8]–[11]), epilepsy (ClC-2 [12]–[14]), Dent's disease (ClC-5 [15]–[19]), and osteopetrosis (ClC-7 [20]). ClC-Kb is expressed in the thick ascending limb of the kidney, where it facilitates chloride reabsorption [21]. ClC-Kb, together with the nearly identical homolog ClC-Ka, serves a similar role in chloride homeostasis in the stria vascularis of the inner ear [22].

**Figure 1:**
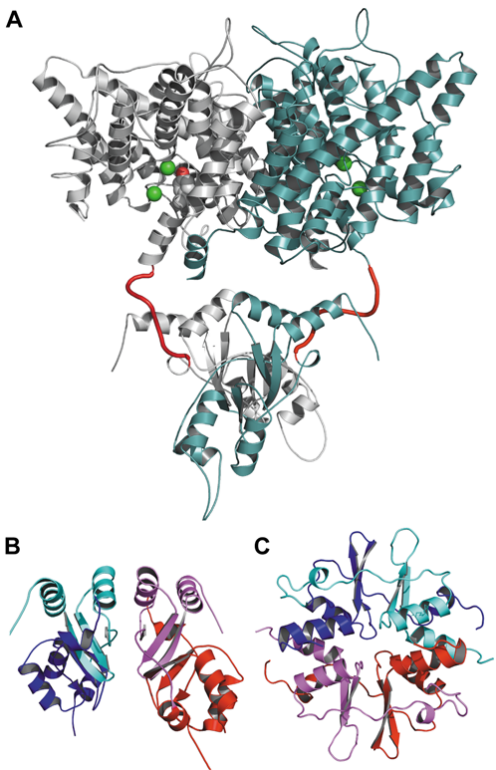
CLC domain architecture and the location of R538. (A) Homology model of ClC-Kb using ClC-ec1 (1OTS) as a template for the membrane domain and the ClC-Ka C-terminal domain structure (2PFI) as a template for the cytoplasmic domain. Subunit A (grey) has several amino acids removed to show the ion coordination of chloride by Y520 (in spacefill), located on helix-R. R538 is located in the cytoplasmic-domain loop (red) that connects helix-R to CBSD1. (B) Structure of the ClC-Ka C-terminal domain (2PFI) highlighting the different CBSDs. Subunit 1 has CBSD1 in blue and CBSD2 in cyan; subunit 2 has CBSD1 in red and CBSD2 in violet. (C) Structure of TM0935 (1OTS) with the color scheme as in B. All cartoons were generated in PyMol (http://www.pymol.org).

Eukaryotic CLC proteins consist of two domains: a membrane domain that forms the chloride-selective permeation pathway and a C-terminal cytoplasmic domain. (Figure 1) [1], [2], [23]. In contrast to the relatively much-studied permeation pathway, the functional role of CLC cytoplasmic domains remains less well understood. Even so, their physiological importance is underscored by the large number of truncations and point mutations that result in disease [24]. While disruption of the cytoplasmic domain can interfere with folding and/or trafficking and thus result in a decrease of functional protein at the plasma membrane [20], [24]–[31], there are clues that the cytoplasmic domains are also important for electrophysiological function. This has most clearly been shown for the skeletal muscle homologs ClC-0 and ClC-1. In ClC-0, mutations in the cytoplasmic domain affect the “slow” cooperative gating [24], [28], [32]. In ClC-1, point mutations in the cytoplasmic domains cause shifts in voltage-dependent gating that are linked to dominantly inherited myotonia [8], [33]. Disease-causing cytoplasmic domain point mutations are also found in several other homologs; however, in these cases, the electrophysiological effects of the mutations are either controversial (ClC-2) [12], [34] or not yet reported (ClC-6, ClC-7, and ClC-Kb) [35]–[37].

Here we study the point mutation R538P, which occurs in the C-terminal cytoplasmic domain of ClC-Kb and causes Bartter's Syndrome [37]. R538 is located in the linker region between the membrane domain and the cytoplasmic domain (Figure 1A). We show that this mutation changes gating at hyperpolarized membrane potentials, alters selectivity and removes activation induced by extracellular calcium. Thus, the mutation of a single amino acid in the cytoplasmic domain causes a long range conformational change that traverses the membrane-spanning domain of ClC-Kb. In the homologous ClC-Ka (90% identical), the R538P mutation does not yield the same changes in gating or calcium activation. This surprising difference indicates that ClC-Ka and ClC-Kb differ either structurally or in how conformational changes are translated to the extracellular domain.

The C-terminal domains of CLCs contain a pair of conserved protein motifs referred to as cystathionine beta-synthase domains (CBSDs) [38]. CBSDs are present in a diverse array of proteins, where they are independent domains that play roles in the regulation of the core domain's catalytic activity [39]. In cystathionine beta-synthase, mutations in the CBSDs eliminate the protein's ability to be activated by endogenous ligand and lead to the disease homocystinuria, a defect in the methionine metabolism pathway [40]. In AMP-activated protein kinase, mutations in the CBSDs of the gamma subunit result in familial hypertrophic cadiomyopathy, presumably because altered regulation of the other subunits [41].

The structures of two human CLC C-terminal cytoplasmic domains, ClC-Ka [42] and ClC-5 [43], have been determined. Both of these constructs form dimers, with the intermolecular interface occurring between CBSD2 of each subunit (Figure 1B). This architecture is preserved in solution, as illustrated by cross-linking studies on the ClC-Ka C-terminal domains [42]. In contrast, a cytoplasmic domain construct from the *Torpedo* homolog ClC-0 crystallizes as a monomer [44]. However, the domain in solution is a dimer, and cross-linking studies on full-length ClC-0 indicates that the dimer interface is similar to that for ClC-Ka and ClC-5 [44].

Despite the similarities between the CBSD structures described above, there is some plasticity in the oligomeric architecture of CBSDs. In IMPDH, for example, the structure of the full-length protein reveals a monomeric CBSD [45]. In the prokaryotic protein TM0935, a protein comprised entirely of CBSDs, the crystal structure (PDB ID 1O50) reveals a dimeric protein that has a completely different intermolecular interface than that observed in ClC-Ka and ClC-5 [52] (Figure 1C). This interface is also observed in a recombinant protein consisting of two of the CBSDs of the eukaryotic protein AMPK [46], and in TA0289, a Thermoplasma acidophilum protein containing a C-terminus Zn ribbon domain along with its CBSD [47].

Because ClC-Kb differs from ClC-Ka in some of the key residues occurring at the dimer interface, we hypothesized that the differences in the effect of R583P on ClC-Ka and ClC-Kb function may reflect differences in the oligomeric architecture of the cytoplasmic domains. On testing this hypothesis, we found that the cytoplasmic domain of ClC-Kb is dimeric and that the dimer interface adopted is similar to that observed in the structure of the C-terminal domain of ClC-Ka.

## Results

### Characterization of the R538P mutation in ClC-Kb

**Figure 2:**
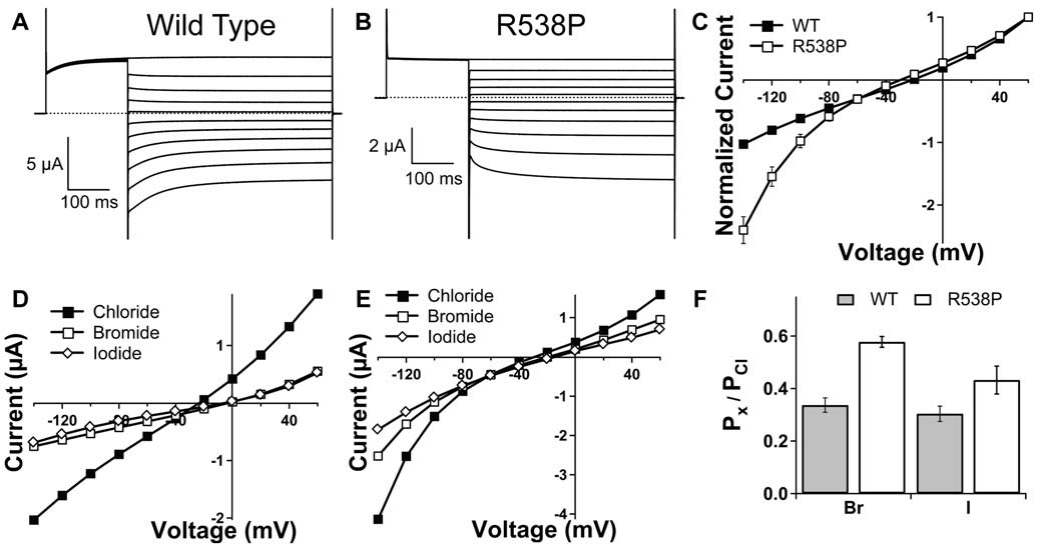
Gating and selectivity of ClC-Kb-R538P. Two-electrode voltage clamp recordings of (A) wild type and (B) R538P ClC-Kb channels. Currents are in response to a 200-ms prepulse to +60 mV followed by 500-ms test pulses ranging from +60 mV to −140 mV in −20 mV decrements. (C) Steady-state currents at the different voltages were normalized to the current value at +60 mV for wild type (filled squares) and R538P (open squares). Error bars are s.e.m. with n = 8 (WT) and n = 6 (R538P). Error bars smaller than the symbols are not shown. Representative steady state I–V curves for (D) wild type and (E) R538P channels in the presence of chloride (filled squares), bromide (open squares), or iodide (open diamonds). (F) Permeability ratios relative to chloride for wild type (filled bars) and R538P (open bars). The bromide/chloride permeability ratio of R538P is statistically different from that of wild type (*p*<0.0005, n = 5).

In ClC-Kb, R538 is located after the last membrane helix (helix-R) immediately preceding CBSD1 (Figure 1). Since helix-R lines the chloride permeation pathway [48], [49] and extends into the cytoplasm, we hypothesized that the R538P mutation could alter helix orientation, thus potentially affecting both gating and permeation. Indeed, we observe alterations in both properties. Wild-type ClC-Kb has a linear steady-state voltage dependence with only slight activation and inactivation at depolarized and hyperpolarized potentials respectively (Figure 2A, C). In contrast, ClC-Kb-R538P is slightly inwardly rectifying and exhibits activation at hyperpolarized potentials (Figure 2B, C). To test whether there are any alterations in the permeation pathway of ClC-Kb-R538P channels, we assessed the relative permeability of different anions. Wild-type ClC-Kb channels show a selectivity sequence of Cl^−^>Br^−^ = I^−^, whereas ClC-Kb-R538P shows a slight but significant increase in the relative permeability of bromide (Figure 2D–F).

**Figure 3:**
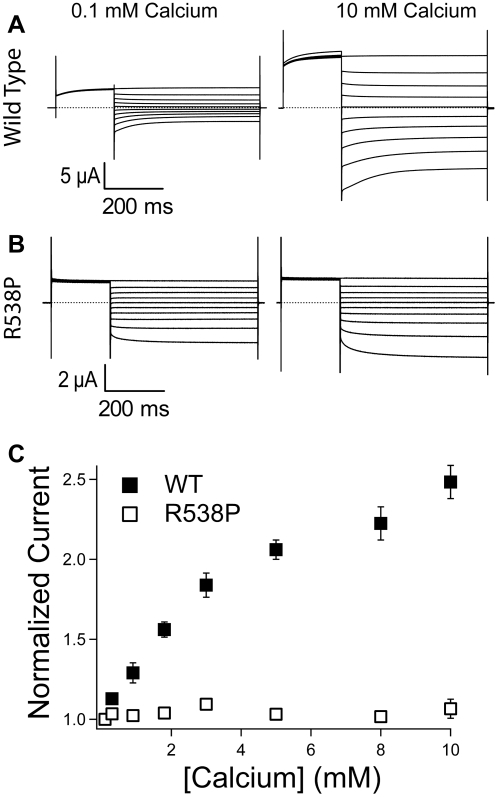
R538P abolishes calcium activation in hClC-Kb. Current recordings for (A) wild type and (B) R538P ClC-Kb channels at 0.1 mM calcium (left) and 10.0 mM calcium (right). (C) Calcium dependent activation of wild type (filled squares) and R538P (open squares) channels. Steady-state current levels at +60 mV plotted as a function of [Ca^2+^], normalized to the value at 0.1 mM calcium. Each data point is the average of several (n = 4–6) oocytes and error bars show the s.e.m. Only oocytes that showed reversibility were included in the analysis.

Two characteristic features of ClC-K channels are their activation by extracellular alkalinization and by extracellular Ca^2+^ [21], [50], [51]. No change was observed in the pH dependence of ClC-Kb-R538P relative to wild type channels (data not shown). In contrast, extracellular activation by Ca^2+^ was abolished: wild type ClC-Kb exhibits a ∼2-fold activation when extracellular Ca^2+^ is raised from 0.1 to 10 mM calcium, whereas no significant activation is seen with ClC-Kb-R538P under the same conditions (Figure 3).

### Characterization of the R538P mutation in ClC-Ka

**Figure 4:**
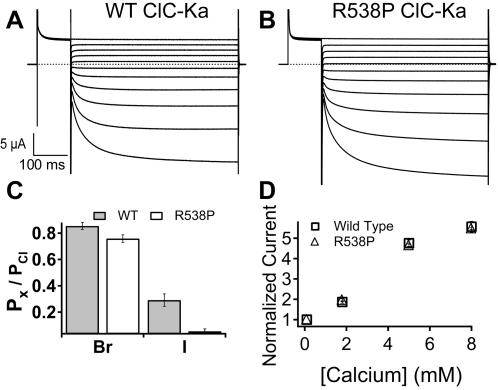
Expression of hClC-Ka-R538P. Two-electrode voltage clamp current recordings of wild type (A) and R538P (B) expressing channels. Currents are in response to a 100-ms prepulse to +60 mV follow by 500-ms test pulses ranging from +60 mV to −140 mV in −20 mV steps. (C) Relative permeability ratios for ClC-Ka wild type (filled bars) and R538P (open bars) channels. The iodide/chloride permeability ratio of R538P is statistically different from that of wild type (*p*<0.02, n = 3 (WT) and n = 5 (R538P)). (D) Calcium dependence at +60 mV of wild type (open squares) and R538P (open triangles) normalized to the current at +60 mV in the presence of 0.1 mM calcium, as in Figure 3. Error bars for WT (n = 4) and R538P (n = 3) are the s.e.m.

Since ClC-Kb shares a high degree of sequence identity with ClC-Ka (∼90% identity), we investigated whether the R538P mutation in ClC-Ka would induce similar changes in voltage dependence, selectivity and calcium activation. Surprisingly, we found only subtle changes: selectivity measurements showed a slight decrease in relative iodide permeation, while voltage- and Ca^2+^ -activation were unaffected (Figure 4). These results suggest that despite the overall similarity between ClC-Ka and ClC-Kb, the properties imparted to the pore by the cytoplasmic domains are quite different.

### Analysis of the ClC-Kb cytoplasmic domain

**Figure 5:**
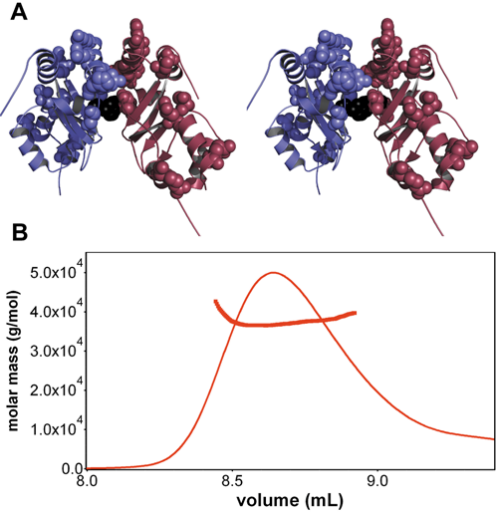
Dimerization of the ClC-Kb C-terminal domain. (A) Stereo view of the ClC-Ka cytoplasmic domain structure (2PFI) with amino acid differences between ClC-Ka and ClC-Kb shown in space fill. Amino acid F636 (shown in black) is on the dimer interface in ClC-Ka and when mutated to aspartic acid removes ClC-Ka dimerization. In ClC-Kb this residue is the non-polar residue serine. (B) Gel filtration combined with multi-angle light scattering shows that the C-terminal domain of ClC-Kb forms a dimer in solution. The solid line represents the absorbance at 280 nm as a function of elution volume. Filled circles represent the calculated mass at the different elution volumes. The molar mass was determined to be 37 kDa, consistent with dimerization.

We hypothesized that the differences in the functional effects of the mutation in ClC-Ka and ClC-Kb might be due to a difference between the two C-terminal cytoplasmic domains. The cytoplasmic domain of ClC-Kb is over 90% identical to the cytoplasmic domain of ClC-Ka, for which a crystal structure has been determined [42], and therefore the structures are likely to be identical. However, when the residues that differ between the two homologs are mapped onto the ClC-Ka crystal structure, it is surprising to note that two of these residues occur at the dimer interface, at the core of the protein. In particular, amino acid 636 is a phenylalanine in ClC-Ka which when mutated removes ClC-Ka cytoplasmic domain dimerization [42]; in ClC-Kb, amino acid 636 is a non-hydrophobic serine (Figure 5A). This suggests the possibility that the ClC-Kb C-terminal domain may not dimerize or dimerize differently than ClC-Ka. We tested this hypothesis by determining the oligomeric status of a recombinant protein corresponding to the C-terminal domain of ClC-Kb. Gel-filtration chromatography and multi-angle light-scattering both indicate that this protein, like that of the C-terminal domain of ClC-Ka, is a dimer in solution (Figure 5B).

**Figure 6:**
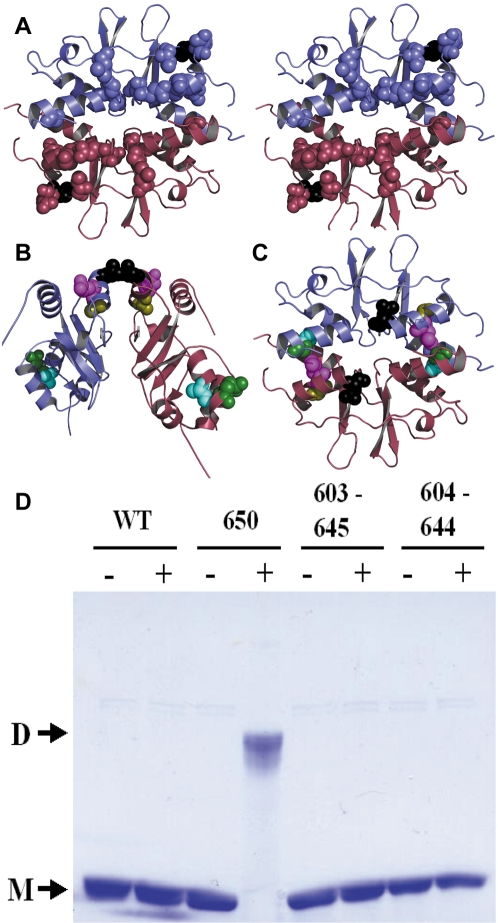
Conserved dimerization interface in the ClC-Kb C-terminal domain. (A) Stereo view of TM0935 structure (1O50), a CBSD-containing protein that adopts an alternate dimer interface. Amino acids corresponding to residues that differ between ClC-Ka and ClC-Kb are shown in spacefill. The residue corresponding to 636 in ClC-Kb (see Figure 5A) is shown in black. (B) Residues chosen for cross-linking studies mapped onto the ClC-Ka C-terminal domain structure: L650 (black), 603 (cyan), 604 (green), 644 (olive), 645 (magenta). For this dimer interface, the L650C mutant is expected to form an intersubunit cross-link. (C) Residues chosen for cross-linking studies mapped onto the TM0935 structure; color scheme as in B. For this dimer interface, the 603–645 and the 604–644 cysteine double mutants are expected to form intersubunit cross-links. (D) SDS-PAGE of purified ClC-Kb C-terminal domains with different cysteine mutations in reducing (−) and oxidizing (+) conditions.

Following this observation, we next sought to compare the dimerization interfaces of the ClC-Ka and Kb cytoplasmic domains. In contrast to the dimerization interface seen in the ClC-Ka crystal structure, a second dimerization interface has been observed in other CBSD-containing proteins, TM0935, TA0289, and AMPK [46], [47], [52]. A number of the residues differing between ClC-Ka and ClC-Kb localize to the helices that form this interface (Figure 6A), which led us to question whether the ClC-Kb cytoplasmic domain might form an interface similar to that observed in TM0935 rather than ClC-Ka. To evaluate the ClC-Kb dimer interface, we used a cysteine-crosslinking strategy. In the ClC-Ka C-terminal domain structure, leucine 650 of one subunit is in close proximity (<5Å) to the same residue in the other subunit, and constructs with the L650C mutation spontaneously cross-link in solution [42]. In the ClC-Kb C-terminal domain protein with the L650C mutation, spontaneous cross-linking in solution is also observed (Figure 6D). Thus, the interface seen in the ClC-Ka C-terminal domain crystal structure likely occurs in ClC-Kb as well.

While the ClC-Kb-L650C crosslink suggests a dimer interface similar to that observed in ClC-Ka, it does not rule out that in ClC-Kb there is an equilibrium between two dimerization conformations: one corresponding to the ClC-Ka dimer and the other to the TM0935 dimer. Such an equilibrium would suggest a “subunit swapping” mechanism, where the cytoplasmic domain interconverts between conformations with different dimerization interfaces. To assess this possibility, we designed two pairs of cysteine mutations that would be predicted to form intersubunit cysteine cross-links based on the TM0935 structure but not on the ClC-Ka structure (Figure 6B, C). ClC-Kb C-terminal domain constructs containing the double mutants K604C-A644C and L603C-H645C both form monodispersed dimers, as observed with wild-type (data not shown). Unlike the L650C mutant, in neither case was spontaneous cross-linking observed (Figure 6D). Moreover, application of H_2_O_2_ to catalyze disulfide-bond formation did not yield cross-links (data not shown). Taken together, these results suggest that ClC-Kb adopts the same dimerization interface as ClC-Ka [42].

## Discussion

Mutation of residue 538 in the C-terminal cytoplasmic domain of ClC-Kb gives rise to Bartter's syndrome [7]. Although the mechanism by which the disease is caused is unknown, we hypothesized based on this residue's location in the cytoplasmic domain that it may affect function. Indeed, ClC-Kb-R538P exhibits slightly altered bromide permeability relative to wild type, indicating that the mutation induces a change in the permeation pathway. Strikingly, R538P removes extracellular calcium activation. Extracellular calcium activation must result in a conformational change that is transmitted from the calcium binding site to the pore. In order for R538P to abolish the activation, it must prevent calcium from binding or disrupt the calcium induced conformational change being transmitted to the pore.

It is not known whether any of the functional changes observed in ClC-Kb-R538P are related to the Bartter's Syndrome phenotype reported by Konrad et al [7]. ClC-Kb is expressed in the basolateral membrane of kidney epithelia where the membrane potential is around −70 mV. While ClC-Kb-R538P exhibits a greater degree of inward rectification compared to wild type, the current-voltage plots show that deviation from wild type behavior is minimal at −70 mV (Figure 2). At the normal basolateral membrane potential, it is not likely that the change in voltage-dependence observed in ClC-Kb-R538P could alone be responsible for the disease phenotype.

The disruption in Ca^2+^ dependence may be responsible for the Bartter's syndrome caused by the R538P mutation. Although not definitive, there are several reports in the literature that support this hypothesis. Ca^2+^ levels are known to affect chloride transport in the thin ascending limb [53], and *in vivo* studies of rat kidney have revealed variations in interstitial Ca^2+^ of about 1 mM [54], in the range where ClC-Kb activity is affected. Moreover, disruption of basolateral calcium receptors in the kidney results in Bartter's-like symptoms [55], [56], which suggests a possible role for calcium fluctuation in the interstitial spaces in the kidney. Together, these results suggest that the loss of Ca^2+^ activation in the ClC-Kb R538P mutant may be responsible for the disease. Nevertheless, there are other factors that cannot be ruled out. It is possible that ClC-Kb-R538P does not traffic properly to the basolateral membrane or that the single-channel conductance is significantly altered, either of which could lead to altered basolateral chloride conductance. When equal amounts of wild-type and mutant ClC-Kb RNA are injected into the same batch of oocytes, there is only a modest (∼50%) difference between wild-type and mutant currents levels (in low Ca^2+^). This suggests that expression level, trafficking, and single-channel conductance are similar. However, it is still possible that such a small difference could be responsible for the disease phenotype. Additionally, it is possible that the difference in expression or trafficking is even greater in the native tissue, and so further studies are necessary to determine the cause of the disease.

CLC C-terminal cytoplasmic domains are required for CLC channel function [24], [26]–[28]. Numerous missense mutations and truncations in these domains result in disease, with many of the mutations occurring in the CBSDs [8], [57], [58]. CBSDs occur in a wide variety of functionally unrelated proteins. As observed in the CLCs, mutations in CBSDs in some of these other proteins lead to disease [39]. In all cases where CBSDs are known to regulate protein function, the CBSDs are structurally distant from the catalytic domain of the proteins (for CLCs, the chloride permeation pathway). Hence, there must be a mechanism for changes in CBSDs to be passed along to the catalytic domain of the proteins to which they are attached.

Surprisingly, we did not observe any changes in calcium activation when R538P was introduced into ClC-Ka. This was surprising, since the ClC-Ka and ClC-Kb channels are very similar (∼90% identical). One possible explanation for the different behaviors is that the structures of the ClC-Ka and ClC-Kb cytoplasmic domains are significantly different, despite their sequence similarity. Supporting this hypothesis is the observation that the majority of amino acids in ClC-Kb that differ from ClC-Ka occur in the residues that would form the two potential dimerization interfaces (the dimerization interface observed in the ClC-Ka C-terminal domain structure and that observed in the prokaryotic CBSD protein TM0935). However, our cross-linking studies indicate that dimer conformation adopted by the ClC-Kb C-terminal domain is that observed in the ClC-Ka structure rather than that of TM0935 and other proteins. Hence the mechanism by which the R538P mutation abolishes Ca^2+^ activation is not likely to involve changes in the dimer interface.

The location of amino acid 538 suggests possible mechanisms by which such a large conformational change can be transmitted through the membrane domain. Helix-R may extend up to or beyond position 538, and the introduction of a proline may result in helix instability (bend or break) that results in a loss of communication between this helix and the pore/extracellular domain. It is also possible that R538P disrupts a direct interaction with the membrane domain or other amino acids in the cytoplasmic domain (either the N-terminus or C-terminus). Testing these hypotheses will require structural studies on full-length protein.

## Materials and Methods

### Molecular biology

Human ClC-Ka, ClC-Kb and Barttin were subcloned into the HindIII and EcoRI sites of psGEM. psGEM constructs were linearized with NheI and transcribed using mMessage T7 transcription kit (Ambion). ClC-Kb cytoplasmic-domain constructs containing amino acids 538–684 were inserted into the EcorR1 and XbaI sites of the pMAL-c2x vector (NEB) to form a C-terminal fusion protein with maltose-binding protein. All mutations were generated using PCR based site-directed mutagenesis.

### Electrophysiology

Defolliculated Xenopus oocytes were injected with 55.6 nL of a 2∶1 mixture of ClC-Kb∶Barttin-Y98A RNA at 550 ng/µL or 27.5 nL of a 2∶1 mixture of ClC-Ka∶Bartin-Y98A RNA at 5–550 ng/µL. (For these studies, the point mutation Y98A in Barttin, which increases surface expression of ClC-K channels [59], was used.) Oocytes were incubated at 17°C for 1–7 days before recording using two-electrode voltage clamp. Electrodes were pulled to 0.4–1.6 MΩ and filled with 3 M KCl, 1.6 mM EGTA, 5 mM HEPES, pH 6.5. The standard bath solution was 96 mM NaCl, 1.8 mM CaCl_2_, 2 mM KCl, 1 mM MgCl_2_, and 5 mM Tris-Cl at pH 8.4. For selectivity experiments, 80 mM chloride was replaced with the indicated anion. Statistical significance was determined using a student t-test. For calcium experiments, CaCl_2_ was excluded and calcium acetate was added to reach the desired calcium concentration.

### Expression and purification of ClC-Kb cytoplasmic domains

ClC-Kb cytoplasmic domains were expressed as C-terminal maltose-binding protein fusions (MBP-KbC). *E. coli* transformed with MBP-KbC were grown at 37°C in LB media to OD_600_ of ∼0.5, transferred to 25°C, induced with 0.3 mM IPTG, and grown for 5 additional hours. Cells were harvested by centrifugation, and pellets were suspended in 50 mL storage buffer (200 mM NaCl, 20 mM Tris-Cl pH 7.4, 1 mM EDTA, 20% glycerol) and frozen overnight or stored at −20°C. Cells were thawed and lysed by sonication after application of protease inhibitor cocktail. Cell lysates were centrifuged for 60′ @ 96,000×*g*. The supernatant was loaded onto an amylose column (NEB), equilibrated with column buffer (200 mM NaCl, 20 mM Tris-Cl pH 7.4, 1 mM EDTA), and protein was eluted with 10 mM maltose in column buffer. MBP-KbC fusion was digested with Factor Xa overnight at 4°C (8–10 hours). The digestion reaction was diluted 10× into cation-exchange buffer A (6 mM NaCl, 20 mM sodium phosphate, pH 6.0) and loaded onto a POROS strong cation-exchange column (Applied Biosystems). After washing away MBP with cation-exchange buffer B (25 mM NaCl, 20 mM sodium phosphate pH 6.0), purified ClC-Kb C-terminal domain (KbC) was eluted with a 25 mM to 500 mM NaCl gradient (in sodium phosphate, pH 6.0).

For cysteine cross-linking experiments, purified KbC was buffer exchanged (using gel-filtration chromatography) into TBS7.5 (200 mM NaCl, 10 mM Tris pH 7.5, 5 mM ß-mercaptoethanol). Samples were reduced with 250 mM ß-mercaptoethanol.

### Multiangle light scattering

A Shodex PROTEIN KW-802.5 size exclusion chromatography column, equilibrated with running buffer (200 mM NaCl, 20 mM Tris-Cl, pH 7.5) was coupled with in-line DAWN EOS multiangle light scattering, refractive index (Wyatt Technology Corporation) and UV (Jasco Corporation) detectors (SEC-MALLS). Monomeric bovine serum albumin at 1 mg/mL was used to calibrate light scattering detectors. Protein at 1 mg/mL was run through the size exclusion column at room temperature (23°C). The molecular weight of the eluted species was calculated using the ASTRA 4.90.08 software provided by Wyatt Technology Corporation.
